# Mutations in *OSBPL2* cause hearing loss associated with primary cilia defects via sonic hedgehog signaling

**DOI:** 10.1172/jci.insight.149626

**Published:** 2022-02-22

**Authors:** Hairong Shi, Hongshun Wang, Cheng Zhang, Yajie Lu, Jun Yao, Zhibin Chen, Guangqian Xing, Qinjun Wei, Xin Cao

**Affiliations:** 1 Department of Medical Genetics, School of Basic Medical Science,; 2 Jiangsu Key Laboratory of Xenotransplantation,; 3 Department of Otolaryngology, The First Affiliated Hospital, Nanjing Medical University, Nanjing, China.

**Keywords:** Genetics, Otology, Cytoskeleton, Genetic variation

## Abstract

Defective primary cilia cause a range of diseases called ciliopathies, which include hearing loss (HL). Variants in the human oxysterol-binding protein like 2 (OSBPL2/ORP2) are responsible for autosomal dominant nonsyndromic HL (DFNA67). However, the pathogenesis of *OSBPL2* deficiency has not been fully elucidated. In this study, we show that the *Osbpl2*-KO mice exhibited progressive HL and abnormal cochlear development with defective cilia. Further research revealed that OSBPL2 was located at the base of the kinocilia in hair cells (HCs) and primary cilia in supporting cells (SCs) and functioned in the maintenance of ciliogenesis by regulating the homeostasis of PI(4,5)P_2_ (phosphatidylinositol 4,5-bisphosphate) on the cilia membrane. OSBPL2 deficiency led to a significant increase of PI(4,5)P_2_ on the cilia membrane, which could be partially rescued by the overexpression of INPP5E. In addition, smoothened and GL13, the key molecules in the Sonic Hedgehog (Shh) signaling pathway, were detected to be downregulated in *Osbpl2*-KO HEI-OC1 cells. Our findings revealed that OSBPL2 deficiency resulted in ciliary defects and abnormal Shh signaling transduction in auditory cells, which helped to elucidate the underlying mechanism of OSBPL2 deficiency in HL.

## Graphical abstract

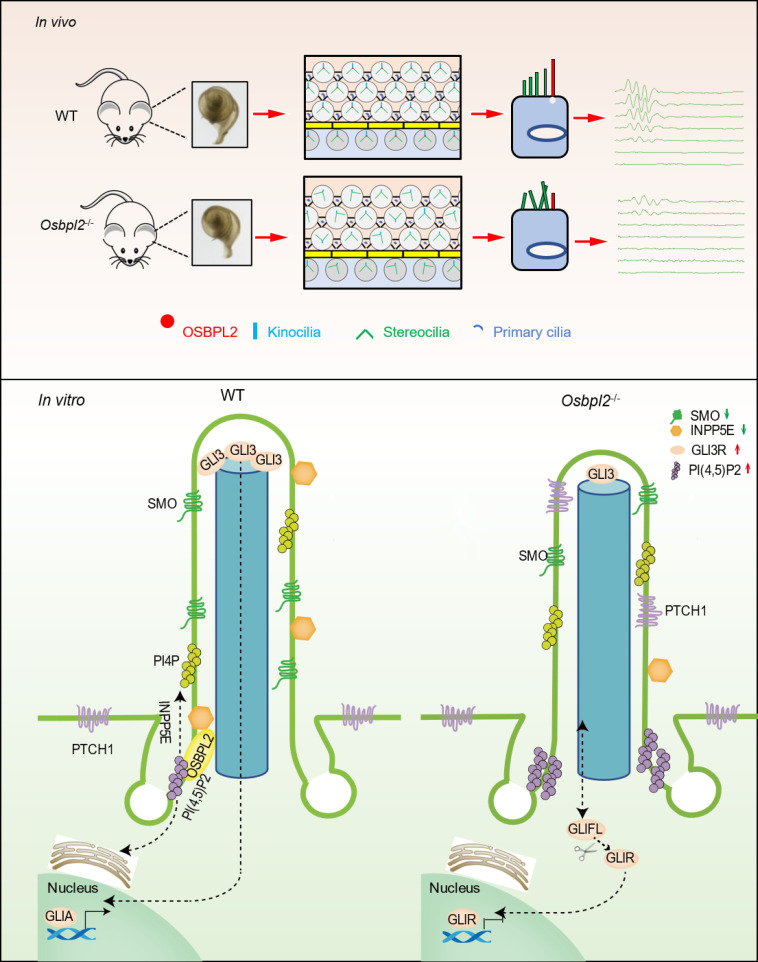

## Introduction

Primary cilia are microtubule-based, antenna-like, and nonmotile organelles that protrude from the apical membrane in many cell types (1–4). Primary cilia that function as mechanosensors and chemosensors participate in various cellular processes, such as signal transduction, cell differentiation, proliferation, and maintenance of stem cells in a wide array of tissues (5–9). Defects in the sensory function or structure of primary cilia are known to be associated with diseases termed ciliopathies (10), which could be caused by genetic defects and implicated in many different organs during embryonic development as well as in the postnatal period.

Auditory hair cells (HCs) are mechanoreceptors of the auditory system and each HC has an apically located hair bundle made up of a single kinocilium and many stereocilia (11). Hearing loss (HL) in relation to HCs’ ciliary defects is also considered to be a kind of ciliopathy (12). To date, studies on cilia-related HL have mainly been focused on stereocilia, which are the key organelles responsible for the conversion of mechanical energy into electrical signals (13–15). Recently, kinocilia have drawn a growing interest due to their important roles in the development of hair bundle polarity and the maintenance of normal hearing (16, 17). Webb et al. reported that the loss of kinociliary links was considered a cause of abnormal polarity of hair bundles in PCDH15-CD2-deficient mice (18). Another study found that genetic defects were implicated in differential defective kinocilia including absence of cilia (*Ift88* mutants), morphologically defective cilia (*Tbc1d32*^bromi^ mutants), or abnormally elongated cilia (*Cilk1* mutants), which resulted in HL in mice (12). However, the molecular mechanism and the key regulators involved in kinociliary development and dynamics have not been thoroughly elucidated.

In our previous study, oxysterol-binding protein like 2 (*OSBPL2*, OMIM: 606731) was identified as a novel deafness-causative gene (DFNA67) in a large Chinese family. Thereafter, the variants in *OSBPL2* were also found to cosegregate with HL in a German family and a Mongolian family (19–21). OSBPL2, also known as ORP2, is a member of the oxysterol-binding proteins-related proteins (OSBP/ORPs) family that participates in various bioprocesses, including cholesterol homeostasis, energy metabolism, cytoskeleton formation, angiogenesis, and signal transduction (22–27). Considering the multiple functions of OSBPL2, the potential pathogenesis of *OSBPL2* deficiency in HL still needed to be further investigated in vitro and in vivo. In auditory cells, we observed that OSBPL2 deficiency promotes ROS production and cell apoptosis (25). In addition, *OSBPL2*-KO pigs displayed progressive HL with degeneration of cochlear HCs and morphological abnormalities in HC stereocilia (28). Interestingly, our recent study found that OSBPL2 was also detected as being localized at the base of the kinocilia, indicating the potential role of OSBPL2 in auditory function associated with the formation of cilia structure and the maintenance of cilia function.

In this study, auditory HEI-OC1 cells and WT mice as well as their *Osbpl2*-KO mutants (*Osbpl2*^–/–^) were used to investigate the cilia-associated mechanism of OSBPL2 deficiency. Our findings showed that *Osbpl2*^–/–^ mice exhibited progressive deafness and demonstrated shortened cochleae and misaligned HCs as well as morphological abnormalities of the kinocilia of HCs. It was found that OSBPL2 was localized at the base of HCs’ kinocilia and SCs’ primary cilia and played an important role in ciliogenesis and Sonic Hedgehog (Shh) signaling transduction in auditory cells by regulating the phosphatidylinositol 4,5-bisphosphate [PI(4,5)P_2_] on the ciliary membrane. Our findings revealed the potential role of OSBPL2 in the regulation of ciliogenesis in auditory cells and helped to elucidate the pathogenesis of *OSBPL2* deficiency implicated in HL.

## Results

### Osbpl2^–/–^ mice showed progressive hearing loss.

**Figure 1:**
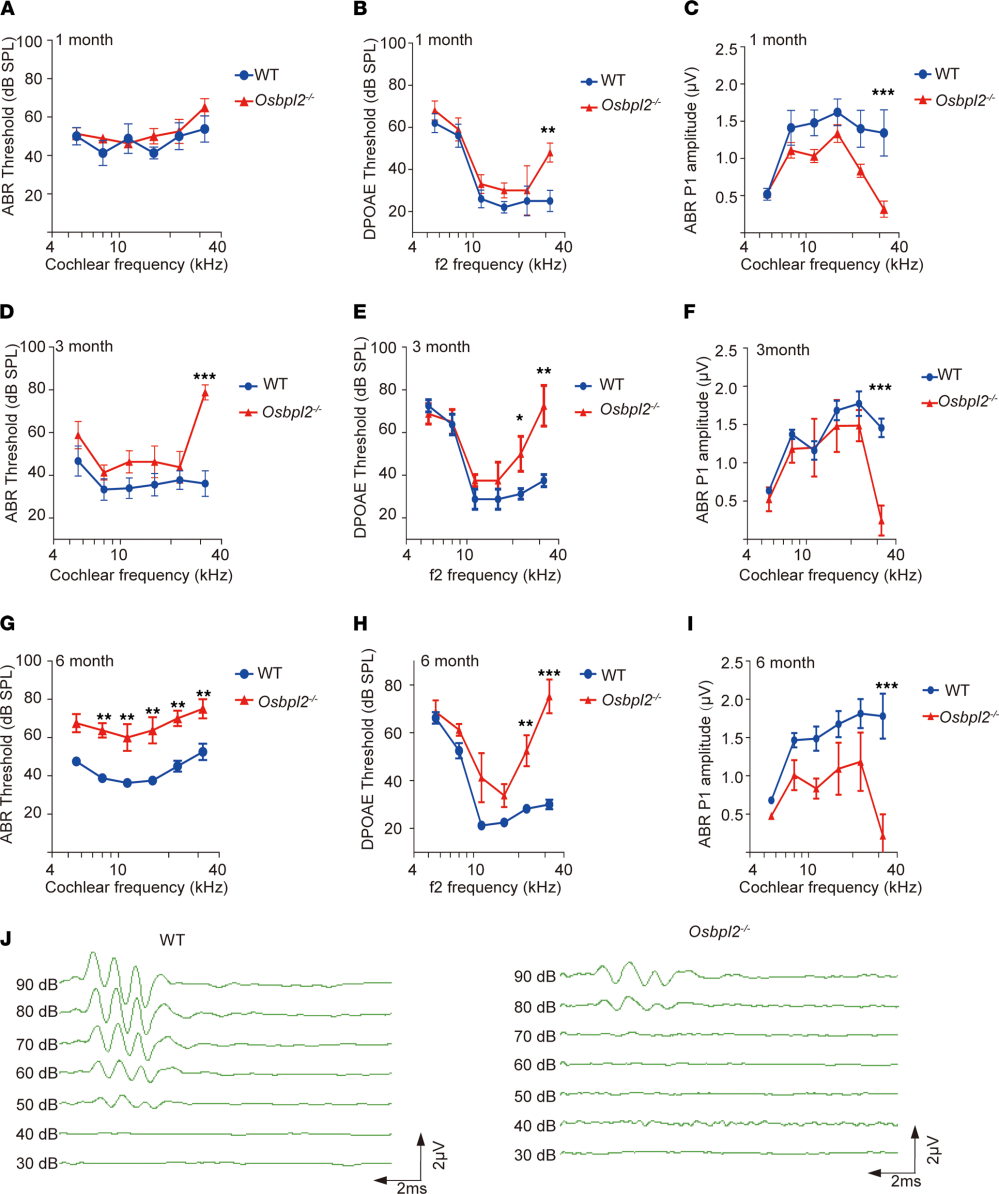
Auditory evaluation in *Osbpl2^–/–^* mice. The ABR thresholds, DPOAE thresholds, and amplitudes of ABR P1 were evaluated in *Osbpl2^–/–^* mice (red, *n* = 6) and age-matched WT controls (blue, *n* = 6). *P < 0.05; **P < 0.01; ***P < 0.001 by 2-tailed Student’s *t* test. (**A**) ABR thresholds (1-month-old), (**B**) DPOAE thresholds (1-month-old), (**C**) amplitudes of ABR P1 (1-month-old), (**D**) ABR thresholds (3-month-old), (**E**) DPOAE thresholds (3-month-old), (**F**) amplitudes of ABR P1 (3-month-old), (**G**) ABR thresholds (6-month-old), (**H**) DPOAE thresholds (6-month-old), and (**I**) amplitudes of ABR P1 (6-month-old). (**J**) ABR waveforms (32 kHz) in 6-month-old *Osbpl2*^–/–^ and WT mice, and the ABR traces were recorded at the same measure range of latency (0–10 ms) and amplitude (0–4 μV).

The *Osbpl2*^–/–^ mouse was constructed using CRISPR/Cas9–mediated gene editing (Supplemental Figure 1, A and B; supplemental material available online with this article; https://doi.org/10.1172/jci.insight.149626DS1), which was confirmed by Western blot analysis, IHC analysis, and genotype analysis (Supplemental Figure 1, C–F). The auditory function of *Osbpl2*^–/–^ 1- to 6-month-old mice was measured by auditory brainstem response (ABR) and distortion product otoacoustic emissions (DPOAEs). At the age of 1 month, there were no remarkable differences of ABR thresholds between *Osbpl2*^–/–^ mice and age-matched WT controls (Figure 1A), while the DPOAE thresholds of *Osbpl2*^–/–^ mice were significantly increased (Figure 1B) and the ABR peak 1 (P1) amplitude of *Osbpl2*^–/–^ mice were significantly reduced at 32 kHz (Figure 1C), indicating that the 1-month-old *Osbpl2*^–/–^ mice had displayed initial HC damage. The 3-month-old *Osbpl2*^–/–^ mice exhibited elevated ABR threshold shifts at each tested frequency and more severe HL was detected at high frequency (Figure 1, D–F). The 6-month-old *Osbpl2*^–/–^ mice maintained hearing decline, which had been aggravated from the high to low cochlear frequencies (Figure 1, G–J). The above results indicated that the *Osbpl2*^–/–^ mice resembled HL and could be used as the disease model of OSBPL2 deficiency.

### Osbpl2^–/–^ mice exhibited defective ciliogenesis and cochlear development.

**Figure 2:**
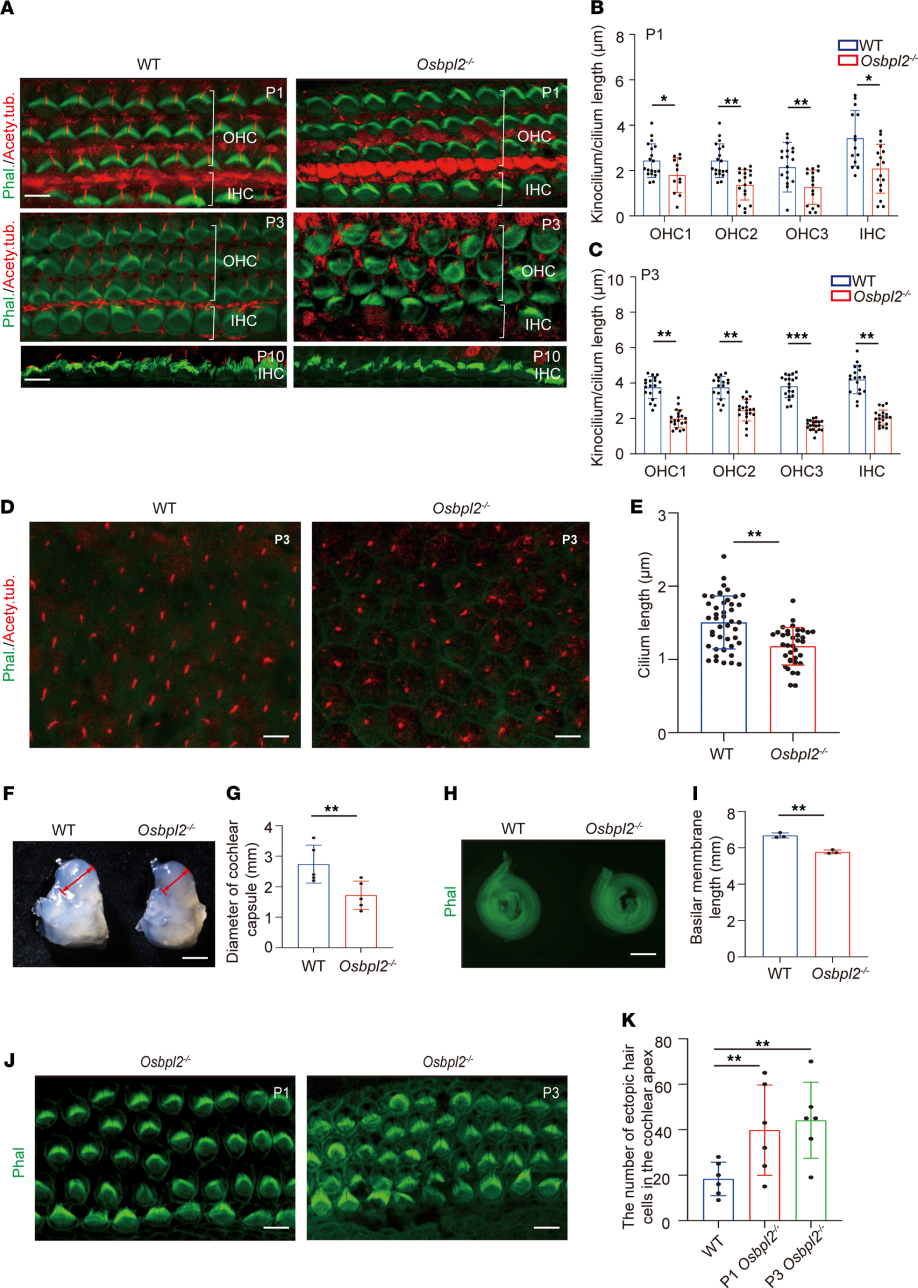
*Osbpl2^–/–^* mice exhibited defective ciliogenesis and abnormal cochlear development. (**A**) Immunofluorescence staining of cilia with anti-acetylated tubulin (red) and stereocilia with phalloidin (green) on the basal turn of sensory epithelium in *Osbpl2*^–/–^ and WT mice (P1, P3, P10). (**B** and **C**) The length of the kinocilia in mouse HCs of P1 and P3 mice (3 mice per genotype, each dot represents a kinocilium; *P < 0.05; **P < 0.01; ***P < 0.001 by 2-tailed Student’s *t* test.) Scale bars: 5 μm. (**D**) Immunofluorescence staining of SCs in cochleae of P3 *Osbpl2*^–/–^ and WT mice. Cilia were stained with anti-acetylated tubulin (red) and cell boundaries are stained with phalloidin (green). (**E**) The length of SC cilia in cochleae of P3 *Osbpl2*^–/–^ and WT mice (3 mice per genotype, each dot represents a cilium; **P < 0.01 by 2-tailed Student’s *t* test). Scale bars: 5 μm. (**F**) Representative images of the cochleae in *Osbpl2*^–/–^ and WT mice (P3). (**G**) The length of otic capsules were analyzed by ImageJ (3 mice per genotype; **P < 0.01 by 2-tailed Student’s *t* test). Scale bars: 100 μm. (**H**) Immunofluorescence images of whole sensory epithelium in *Osbpl2*^–/–^ and WT mice cochleae. (**I**) The length of whole sensory epithelium in *Osbpl2*^–/–^ and WT mice cochleae were analyzed by ImageJ (3 mice per genotype; **P < 0.01 by 2-tailed Student’s *t* test). Scale bars: 500 μm. (**J**) *Osbpl2*^–/–^ mice (P1 and P3) showed 5 rows of HCs versus 4 rows of HCs in WT controls at cochlear apex. Scale bars: 5 μm. (**K**) The number of ectopic HCs in the cochlear apex (6 mice per genotype, each dot represents a mouse cochlea. **P < 0.01 by 1-way ANOVA).

Although the ABR results indicated that the *Osbpl2*^–/–^ mice exhibited late-onset HL, the auditory evaluation via DPOAE and ABR P1 amplitudes indicated that the HC dysfunction had appeared at the early postnatal stage of *Osbpl2*^–/–^ mice. Therefore, 1- to 10-day-old (P1–P10) *Osbpl2*^–/–^ mice were used to explore the pathological basis of OSBPL2 deficiency associated with HL. The bundles’ morphology of cochlear HCs in the *Osbpl2*^–/–^ mice was characterized by staining sensory epithelia with phalloidin and anti-acetyl tubulin. It was observed that kinocilia in the outer HCs (OHCs) and inner HCs (IHCs) and primary cilia in SCs of the cochlear basal turn were shorter in P1 and P3 *Osbpl2*^–/–^ mice than those in WT controls (Figure 2, A–E). Notably, the kinocilia of IHCs of the cochlear base were mostly lost in P10 *Osbpl2*^–/–^ mice (Figure 2A). These results indicated that OSBPL2 deficiency inhibited ciliogenesis in mice cochleae.

It has been reported that ciliary defects could be responsible for cochlear developmental abnormality and stereocilia bundle polarity disorder (29, 30), which were also detected in *Osbpl2*^–/–^ mice. The results showed that the otic capsules of *Osbpl2*^–/–^ mice were observed to be smaller than those of the WT controls (Figure 2, F and G). Meanwhile, the sensory epithelium was shorter in *Osbpl2*^–/–^ mice than that in the control group (Figure 2, H and I). *Osbpl2*^–/–^ mice (P1 and P3) also showed 5 rows of HCs versus 4 rows of HCs in age-matched WT controls at the cochlear apex (Figure 2, J and K), and no ectopic HCs were detected in the middle or base of the cochleae (Supplemental Figure 2A). To evaluate the stereociliary polarity, the hair bundle morphology of HCs in *Osbpl2*^–/–^ mice was characterized by staining with phalloidin. The results indicated that the abnormal polarity of the HCs was detected in P1 and P3 *Osbpl2*^–/–^ mice (Supplemental Figure 2A). In addition, HC polarity was evaluated by measuring the angle between the stereociliary bundles and the mediolateral axis of the epithelium (31) (Supplemental Figure 2B). Some stereocilia bundles were misoriented in the cochlear basal turn of P1 *Osbpl2*^–/–^ mice, which was more significant in P3 *Osbpl2*^–/–^ mice (Supplemental Figure 2C). The above results suggest that OSBPL2 played a crucial role in ciliogenesis, cochlear development, and stereociliary polarity.

### OSBPL2 was localized at the base of cilia and regulated the ciliary length.

**Figure 3:**
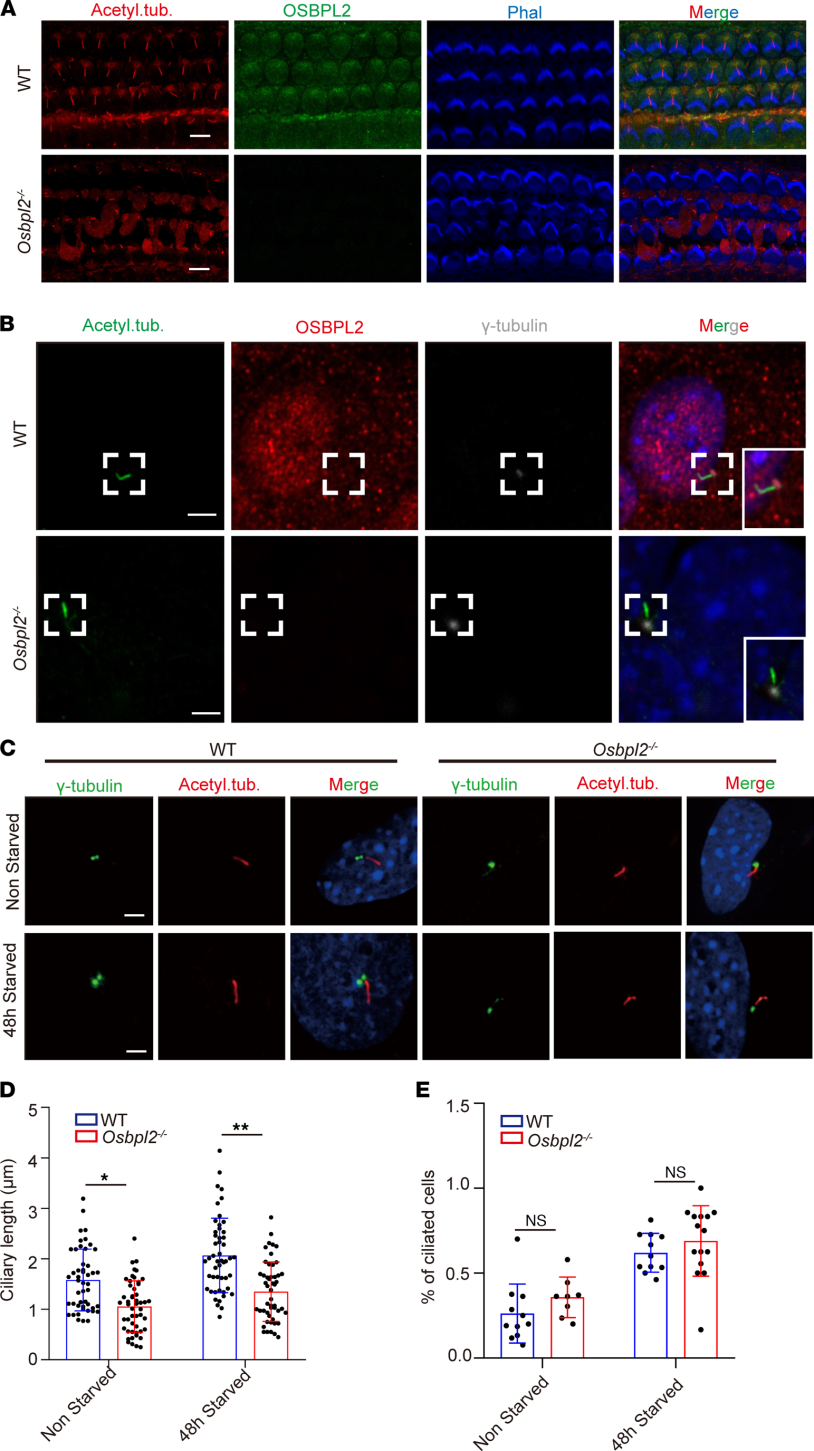
OSBPL2 was localized at the base of cilia and regulated the ciliary length. (**A**) Immunofluorescence staining of the basal turn of sensory epithelium in P3 *Osbpl2*^–/–^ and WT mice with anti-acetylated tubulin (red), anti-OSBPL2 (green), and phalloidin (blue). Scale bars: 5μm. (**B**) Immunofluorescence staining of *Osbpl2*^–/–^ and WT HEI-OC1 cells with anti-acetylated tubulin (green), anti-OSBPL2 (red), anti–gamma-tubulin (grey), and DAPI (blue). Scale bar: 5 μm. (**C**) Immunofluorescence staining of *Osbpl2*^–/–^ and WT HEI-OC1 cells with anti-acetylated tubulin (red in cilia), anti–gamma-tubulin (green in ciliary base bodies), and DAPI (blue in nuclei). (**D**) The length of cilia in *Osbpl2*^–/–^ and WT HEI-OC1 cells (50 cells per genotype, each dot represents a cell; *P < 0.05; **P < 0.01 by 2-tailed Student’s *t* test). (**E**) The proportion of ciliated cells in *Osbpl2*^–/–^ and WT HEI-OC1 cells (50 cells per genotype, each dot represents the proportion of ciliated cells in a microscope field; ns: not significant by 2-tailed Student’s *t* test). Scale bar: 5 μm.

OSBPL2 was detected at the base of the kinocilia of HCs in the cochlear basal turn of WT controls, but not in the *Osbpl2*^–/–^ mice cochleae, which could exclude false positives for antibodies (Figure 3A). Kinocilia in HCs are a specialized type of primary cilia. Therefore, we surmised that OSBPL2 is located at the base of the primary cilia in the same way. To confirm this possibility and investigate how OSBPL2 deficiency causes cilia dysfunction, *Osbpl2*^–/–^ HEI-OC1 cells were established by CRISPR/Cas9 mediated gene editing (Supplemental Figure 3, A–C). The subcellular localization of OSBPL2 in HEI-OC1 cells was investigated and the results showed that OSBPL2 signals were also detected at the ciliary base and overlapped with the gamma-tubulin signals of HEI-OC1 cells (Figure 3B and Supplemental Figure 3D), which was consistent with the results obtained in the mice cochleae HCs. These results indicated that OSBPL2 was localized at the base of cilia and involved in ciliogenesis.

It was noteworthy that *Osbpl2*^–/–^ HEI-OC1 cells cultured in complete medium (not starved) were detected to have shorter primary cilia than WT controls, which was the same with the results of HC kinocilia in *Osbpl2*^–/–^ mice. Given that primary cilia was resorbed during cell-cycle reentry (32), HEI-OC1 cells were cultured in low-serum medium, which ensured that the primary cilia could remain in the growth phase to eliminate the effect of the cell cycle on primary cilia. In HEI-OC1 cells cultured in low serum (48 hours starved), the primary cilia in *Osbpl2*^–/–^ cells were significantly shorter than those in the WT controls (Figure 3, C and D). With or without serum-starved cell culture, the number of ciliated cells showed no significant difference in *Osbpl2*^–/–^ and WT HEI-OC1 cells (Figure 3E). These results indicate that OSBPL2 is essential to the maintenance of primary cilia.

### OSBPL2 regulated ciliogenesis by maintaining the homeostasis of phospholipids on the cilia membrane.

**Figure 4:**
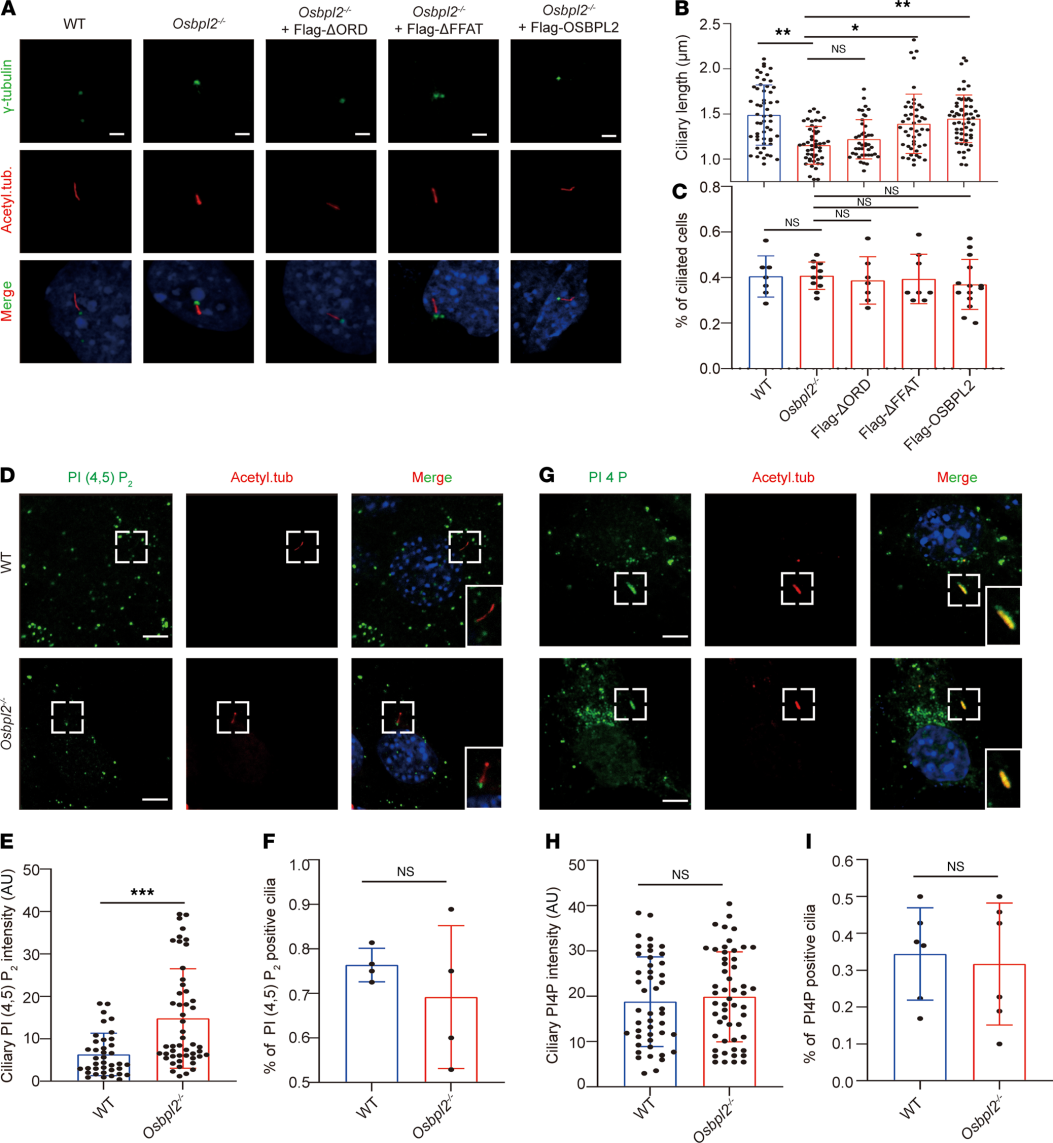
OSBPL2 regulated ciliogenesis by maintaining PI homeostasis on the ciliary membrane. (**A**) Immunofluorescence staining of cilia in *Osbpl2*^–/–^ HEI-OC1 cells transiently expressing Flag-ΔFFAT, Flag-ΔORD, and Flag-OSBPL2. Cells were stained with anti-acetylated tubulin (red), anti–gamma-tubulin (green), and DAPI (blue). Scale bar: 5 μm. (**B**) The ciliary length of *Osbpl2*^–/–^ HEI-OC1 cells transiently expressing Flag-ΔFFAT, Flag-ΔORD, and Flag-OSBPL2 (50 cells per genotype, each dot represents a cell in a microscope field. *P < 0.05; **P < 0.01; ns: not significant; tested by 1-way ANOVA). (**C**) The proportion of ciliated cells in *Osbpl2*^–/–^ HEI-OC1 cells transiently expressing Flag-ΔFFAT, Flag-ΔORD, and Flag-OSBPL2 (each dot represents the proportion of ciliated cells in a microscope field; ns: not significant; tested by 1-way ANOVA). (**D**) Immunofluorescence staining of HEI-OC1 cells with anti-PI(4,5)P_2_ (green), anti-acetylated tubulin (red), and DAPI (blue). Dashed frames denote the locally zoomed regions in solid frames (bottom right). Scale bar: 5 μm. (**E**) Quantification of ciliary PI(4,5)P_2_ intensity in HEI-OC1 cells (50 cells per genotype, each dot represents a cell. ***P < 0.05 by 2-tailed Student’s *t* test). (**F**) The proportion of PI(4,5)P_2_ positive cilia in *Osbpl2*^–/–^ HEI-OC1 cells (each dot represents the proportion of PI(4,5)P_2_ positive cilia in a microscope field. ns: not significant by 2-tailed Student’s *t* test). (**G**) Immunofluorescence staining of HEI-OC1 cells with anti-PI4P (green), anti-acetylated tubulin (red), and DAPI (blue). PI4P showed no significant difference in *Osbpl2*^–/–^ and WT HEI-OC1 cells. Dashed frames denote the locally zoomed regions in solid frames (bottom right). Scale bar: 5 μm. (**H**) Quantification of ciliary PIP4 intensity in HEI-OC1 cells (50 cells per genotype, each dot represents a cell. ns: not significant by 2-tailed Student’s *t* test). (**I**) The proportion of PI4P positive cilia in *Osbpl2*^–/–^ HEI-OC1 cells (each dot represents the proportion of PI4P positive cilia in a microscope field. ns: not significant by 2-tailed Student’s *t* test).

To explore the key domains of OSBPL2 affecting ciliogenesis, the truncated OSBPL2 was constructed. OSBPL2 is one of the short ORPs comprising only 2 domains: 2 phenylalanines in an acidic tract motif (FFAT) and the OSBP-related domain (ORD). The FFAT motif is an ER-targeting residue and the ORD domain refers to a highly conserved region shared by OSBP/ORPs family members, which was the most functional part of OSBPL2 for binding and transferring sterols, oxysterols, and PIs (23, 25, 33). When Flag-OSBPL2 and Flag-ΔFFAT were reexpressed in *Osbpl2*^–/–^ HEI-OC1 cells, the length of primary cilia was restored in Flag-OSBPL2–expressing cells, but not significantly changed in Flag-ΔORD–expressing cells (Figure 4, A and B). In addition, regardless of which truncated protein was reexpressed, the proportion of ciliated cells remained unchanged (Figure 4C). The above results suggest that ORD in OSBPL2 is essential to the regulation of ciliogenesis.

Given that OSBPL2 regulated the distribution of PIs on the cell membrane, which was crucial to the homeostasis of PIs (34), we presumed that OSBPL2 might regulate ciliogenesis by regulating the relative levels of PIs on the ciliary membrane. Compared with the WT controls, PI(4,5)P_2_ was enriched proximal to the axoneme at the cilia base in *Osbpl2*^–/–^ HEI-OC1 cells (Figure 4, D–F). However, PI4P was present along the primary cilia and showed no significant difference between *Osbpl2*^–/–^ HEI-OC1 cells and WT controls (Figure 4, G–I). It was known that the function of inositol polyphosphate-5-phosphatase E (INPP5E) is to catalyze the conversion of PI(4,5)P_2_ to PI4P (35, 36). Our findings showed that OSBPL2 could directly interact with INPP5E by forming a protein complex (Supplemental Figure 4, A and B). Although the protein levels of INPP5E did not change significantly in *Osbpl2*^–/–^ or WT HEI-OC1 cells (Supplemental Figure 4C), a decrease of ciliary INPP5E was detected in *Osbpl2*^–/–^ HEI-OC1 cells (Supplemental Figure 4, D–F). These results may help explain the disturbance of ciliary PIs homeostasis associated with OSBPL2 deficiency.

### OSBPL2 deficiency led to ciliary defects and inhibited the transduction of the Shh signaling pathway.

**Figure 5:**
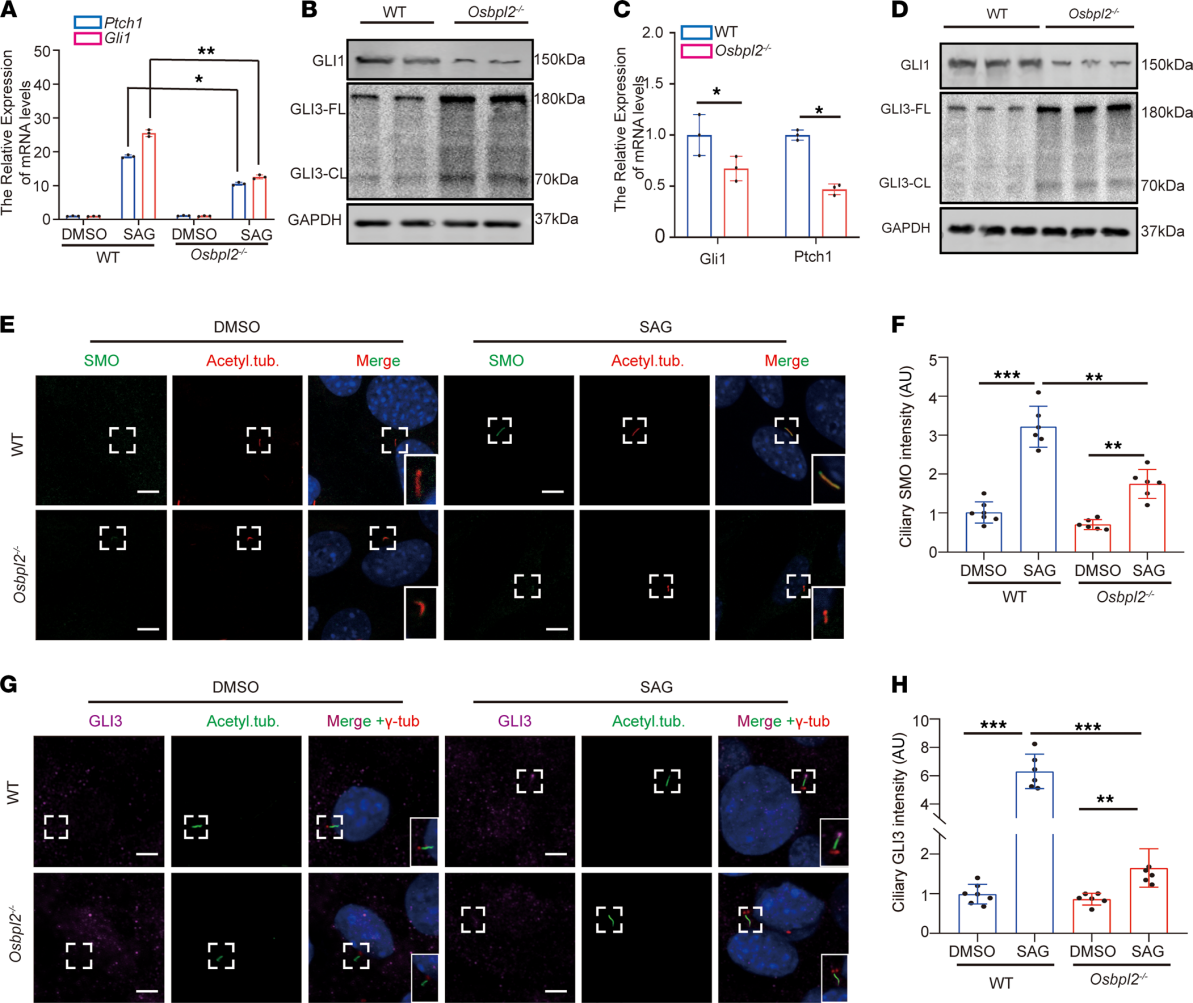
OSBPL2 deficiency inhibited the transduction of the Shh signaling pathway. (**A**) The relative expression of Shh target genes *Ptch1* and *Gli1* in *Osbpl2*^–/–^ and WT HEI-OC1 cells treated with SAG or vehicle (DMSO) (*n* = 3; *P < 0.05; **P < 0.01 by 2-way ANOVA). (**B**) Immunoblotting assay of GLI1 and GLI3 in *Osbpl2*^–/–^ and WT HEI-OC1 cells treated with SAG. (**C**) The relative expression of *Gli1* and *patch1* in cochleae of *Osbpl2*^–/–^ and WT mice at P1 (*n* = 3; *P < 0.05 by 1-way ANOVA). (**D**) Immunoblotting assay of GLI1 and GLI3 in cochleae of *Osbpl2*^–/–^ and WT mice (P1). (**E**) Immunofluorescence staining of *Osbpl2*^–/–^ and WT HEI-OC1 cells with anti-SMO (green), anti-acetylated tubulin (red), and DAPI (blue). Cells were treated with SAG or vehicle (DMSO). Ciliary SMO levels were detected to be significantly increased in *Osbpl2*^–/–^ HEI-OC1 cells. Dashed frames denote the locally zoomed regions in solid frames (bottom right). Scale bar: 5 μm. (**F**) Quantification of ciliary SMO intensity in *Osbpl2*^–/–^ and WT HEI-OC1 cells (at least 6 cells from a microscope field, each dot represents a cell. **P < 0.01; ***P < 0.001 by 2-way ANOVA). (**G**) Immunofluorescence staining of *Osbpl2*^–/–^ and WT HEI-OC1 cells with anti-GLI3 (purple), anti-acetylated tubulin (green), anti–gamma-tubulin (red), and DAPI (blue). Cells were treated with SAG or vehicle (DMSO). Dashed frames denote the locally zoomed regions in solid frames (bottom right). Scale bar: 5 μm. (**H**) Quantification of ciliary GLI3 intensity in *Osbpl2*^–/–^ and WT HEI-OC1 cells (at least 6 cells from a microscope field, each dot represents a cell. **P < 0.01, ***P < 0.001 by 2-way ANOVA).

In the above studies, we elaborated on the mechanism by which OSBPL2 deficiency led to primary cilia defects, but these defects do not account for the developmental abnormality in the cochleae of *Osbpl2*^–/–^ mice, including misaligned HCs and shortened cochleae. Previous studies had reported that ciliary defects resulted in abnormal Shh signal transduction (37, 38). Therefore, it was presumed that the developmental abnormality of the cochlea associated with OSBPL2 deficiency might be attributable to the abnormal Shh signaling pathway. Stimulating HEI-OC1 cells with smoothened (SMO) agonist (SAG) robustly increased transcription of GLI family zinc finger 1 (*Gli1*) and patched 1 (*Ptch1*), both Shh target genes. These responses were attenuated in *Osbpl2*^–/–^ HEI-OC1 cells (Figure 5A), indicating the pivotal role of OSBPL2 in Shh signaling transduction. It was found that GLI3R was upregulated and GLI1 was significantly downregulated in SAG-treated *Osbpl2*^–/–^ HEI-OC1 cells. (Figure 5B). In the mice cochleae, the same results were detected as in HEI-OC1 cells. The mRNA levels of *Gli1* and *Patch1* were decreased in the cochleae of P10 *Osbpl2*^–/–^ mice (Figure 5C). In addition, downregulated GLI1 and upregulated GLI3R were also observed in the cochleae of P10 *Osbpl2*^–/–^ mice (Figure 5D). These results indicate that loss of OSBPL2 function inhibited Shh signal transduction.

The subcellular localization of SMO and GLI3 on cilia was detected by immunofluorescence. The localization of SMO in cilia was rarely observed under basal conditions (DMSO vehicle control). After cells were treated with SAG, decreased levels of ciliary SMO were observed in primary cilia of *Osbpl2*^–/–^ cells. (Figure 5, E and F), indicating that OSBPL2 deficiency significantly reduced SMO accumulation in cilia after Shh pathway activation. It was also found that low levels of ciliary GLI3 were detected in both *Osbpl2*^–/–^ cells and WT controls. After SAG treatment, GLI3 was accumulated at the ciliary tip of control cells, while no significant accumulation of GLI3 was detected at the ciliary tip of *Osbpl2*^–/–^ HEI-OC1 cells (Figure 5, G and H). These results suggest that OSBPL2 was important for the transport of Shh-related proteins in primary cilia and the maintenance of Shh signal transduction.

### Shh signal transduction was rescued by alleviating the accumulation of PI(4,5)P_2_ at the base of the cilia.

**Figure 6:**
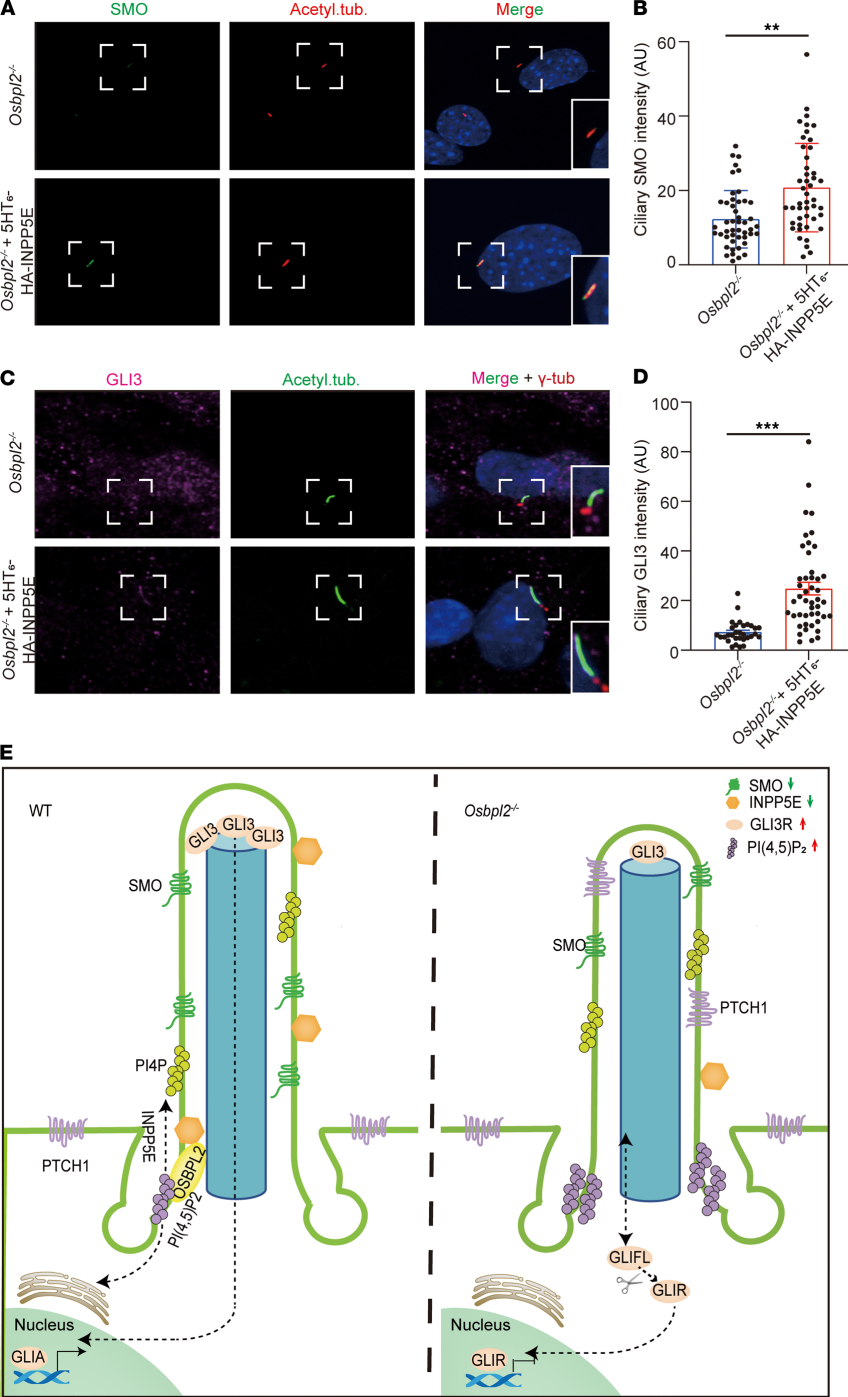
Shh signal transduction was rescued by alleviating the accumulation of PI(4,5)P_2_ at the base of the cilia. (**A**) Immunofluorescence staining of *Osbpl2*^–/–^ HEI-OC1 cells with anti-SMO (green), anti-acetylated tubulin (red), and DAPI (blue). In *Osbpl2*^–/–^ HEI-OC1 cells expressing 5HT_6_-HA-INPP5E, the localization of ciliary SMO was partially rescued. Dashed frames denote the locally zoomed regions in solid frames (bottom right). Scale bar: 5 μm. (**B**) Quantification of ciliary SMO intensity in *Osbpl2*^–/–^ HEI-OC1 cells with or without INPP5E expression (at least 30 cells from a microscope field, each dot represents a cell. **P < 0.01 by 2-tailed Student’s *t* test). (**C**) Immunofluorescence staining of *Osbpl2*^–/–^ HEI-OC1 cells with anti-GLI3 (purple), anti-acetylated tubulin (green), and anti–gamma-tubulin (red). In *Osbpl2*^–/–^ HEI-OC1 cells expressing 5HT_6_-HA-INPP5E, the localization of ciliary GLI3 was partially rescued. Dashed frames denote the locally zoomed regions in solid frames (bottom right). Scale bar: 5 μm. (**D**) Quantification of ciliary GLI3 intensity in *Osbpl2*^–/–^ HEI-OC1 cells with or without INPP5E expression (at least 30 cells from a microscope field, each dot represents a cell; ***P < 0.001 by 2-tailed Student’s *t* test). (**E**) Schematic diagram of OSBPL2 regulating ciliogenesis and Shh signaling transduction in auditory cells. OSBPL2 was localized at the base of the cilia and regulated the homeostasis of ciliary PI(4,5)P_2_, which affected the ciliogenesis and thereby influenced transduction of the Shh signaling pathway. OSBPL2 deficiency led to dyshomeostasis of ciliary PI(4,5)P_2_, which was responsible for ciliary defects and inhibited Shh signal transduction.

Our findings suggest that OSBPL2 deficiency led to the dyshomeostasis of ciliary PI(4,5)P_2_, which was responsible for ciliary defects and abnormal Shh signal transduction. On this basis, it was expected that the abnormal Shh signal transduction caused by OSBPL2 deficiency could be rescued by restoring PI(4,5)P_2_ homeostasis. To express INPP5E in primary cilia, a ciliary localization sequence 5HT_6_ was added at the N-terminus of HA-INPP5E (39). The disruption of PI(4,5)P_2_ on primary cilia was partially restored in *Osbpl2*^–/–^ HEI-OC1 cells which reexpressed INPP5E (Supplemental Figure 5). In a similar vein, we observed that the localization of SMO and GLI3 in the cilia was partially rescued (Figure 6, A–D), indicating that the transduction of the Shh signaling pathway was reactivated. The above results indicate that OSBPL2 deficiency led to ciliary defects with increased cilia PI(4,5)P_2_ and resulted in the inhibition of the Shh signaling pathway, which could be partially rescued by the overexpression of INPP5E.

## Discussion

Primary cilia are implicated in multiple developmental signaling pathways, and they function as chemosensors, mechanosensors, or both, depending on cell type. The structure of cilia can be classified into 3 subdomains: the intracellular basal body (BB), which controls cilium formation; the extracellular axoneme, which generates force; and the transition zone (TZ), which bridges cilia (40, 41). Our findings first revealed that OSBPL2 was localized at the base of the cilium in auditory cells, which encompassed the distal end of the BB and the TZ. The TZ represents a compartment at the base of the primary cilia at the proximal end of the axoneme which, along with the basal body transition fibers, controls ciliary protein entry and exit (42–44). NUP98 was localized at the ciliary base, where it was implicated in the size-exclusion effect of TZ that limited the diffusion of soluble macromolecules into the cilium (45). SEPT2, a member of the septin family of guanosine triphosphatases, has been reported to form a diffusion barrier at the base of the ciliary membrane (46). In addition, identification of a size-exclusion zone at the TZ has raised the possibility of a “lipid gate” barrier that is essential to control the abundance of ciliary PI(4,5)P_2_ (47). In this study, we demonstrated that OSBPL2 was partially localized at the base of the cilia in auditory cells, which functioned in TZ and played an important role in regulating the homeostasis of ciliary PI(4,5)P_2_.

PIs play major roles in regulating many cellular functions, including cell signaling and membrane trafficking. Several OSBP/ORPs family members, including OSBPL2 (ORP2), ORP3, ORP5, and ORP8, have been reported to be associated with the regulation of PIs, suggesting that OSBP/ORPs proteins might have a common role in regulating membrane phosphoinositide composition (34, 48–50). The relationship between OSBPL2 and PIs, e.g., PI(4,5)P_2_, PI4P, and PI(3,4,5)P_3_, on the plasma membrane has been described in previous studies (23). A recent study also reported that OSBPL2 deficiency caused an increase of PI(4,5)P_2_ on the plasma membrane (PM), but PI(4,5)P_2_ on ciliary membrane was not described in detail (34). PIs were also observed to be present around the cilia membrane (51, 52). However, the implication of OSBPL2 in the regulation of ciliary PIs has not been previously elucidated. In this study, we found that OSBPL2 was localized at the base of the cilium and regulated the homeostasis of PI(4,5)P_2_, which could affect the ciliogenesis and, thereby, influence transduction of the ciliary signaling pathway.

The specialized roles of primary cilia were dependent on the mediation of various ciliary signaling pathways including PDGF, GPCRs, RTKs, TGF-β, Notch, WNT, the planar cell polarity (PCP) pathway, and the Shh pathway (5, 53, 54). Among these ciliary signaling pathways, the Shh signaling pathway regulates cell fate and self-renewal in development and tissue homeostasis (1, 2). The importance of Shh in the development of the cochlea has been described in previous studies (55, 56). In the absence of Shh (*Shh^–/–^*), the cochleae were completely damaged. When Shh was impaired due to defects in GLI3, extra rows of HCs and short cochlea were found in the inner ear (57, 58). In this study, *Osbpl2*^–/–^ mice exhibited ciliary defects in the cochleae and inhibited Shh signaling transduction. Indeed, we found that defective GLI activator/repressor functions (GLI1 and GLI3) could help to explain the abnormal cochlear development in *Osbpl2*^–/–^ mice. Our findings were consistent with a previous report that defective GLI activator/repressor functions, short cochlea, and extra rows of HCs were found in the distal region of the cochlea when Shh signaling transduction was impaired (12). In addition, we found that OSBPL2 was essential to SMO accumulation in cilia after activation of the Shh signaling pathway. Importantly, the 5-phosphatase activity of INPP5E, which regulates the levels of PI(4,5)P_2_, was essential to SMO accumulation in cilia as well as Shh signaling transduction. In addition to OSBPL2, several other OSBP/ORPs family members, including ORP1L, ORP3, ORP5, ORP8, and ORP10, have been suggested as being related to the Shh signaling pathway and ciliary function (59–62). In this study, we revealed that OSBPL2 might mediate the Shh signaling pathway by regulating the level of PI(4,5)P_2_ on the ciliary membrane. Our study broadened the functional spectrum of OSBP/ORPs family members and provided the molecular mechanism of OSBPL2 in the regulation of Shh signaling pathway and ciliary function.

In summary, we first described the implication of OSBPL2 in ciliogenesis, cochlea development, and ciliary signaling transduction. It was revealed that OSBPL2 deficiency induced the disturbance of PI homeostasis in the cilia, led to ciliary defects and inhibited Shh signaling transduction in auditory cells (Figure 6E), which could be responsible for progressive HL and abnormal cochleae development in *OSBPL2*^–/–^ mice. Our findings contributed to understanding the pathogenesis of OSBPL2 deficiency and provided the potential molecular mechanism of *OSBPL2* variants associated with human DFNA.

## Methods

### Antibodies and regents.

The antibodies used in this study included anti-acetylated tubulin (Sigma-Aldrich, T7451 and Cell Signaling Technology, 5335); anti-ARL13B (Proteintech, 17711-1-AP); anti-gamma tubulin (Abcam, ab179503); anti-INPP5E (Proteintech, 17797-1-AP); anti-OSBPL2 (Proteintech, 14751-1-AP and Abclonal, A14199); anti-FLAG (Sigma-Aldrich, F1804); anti-HA (Cell Signaling Technology, 3724); anti-GAPDH (Cell Signaling Technology, 5174); anti–PI(4,5)P_2_ (Echelon, Z-P045); anti–PI4P (Echelon, Z-P004); anti-SMO (Santa Cruz, sc-166685); anti-Gli3 (Abcam, ab6050 and Proteintech, 19949-1-AP); anti-Gli1 (Proteintech, 66905-1-Ig); Donkey anti-Mouse IgG (H+L) Highly Cross-Adsorbed Secondary Antibody, Alexa Fluor 555 (Invitrogen, A31570); Donkey anti-Rabbit IgG (H+L) Highly Cross-Adsorbed Secondary Antibody, Alexa Fluor 546 (Invitrogen, A10040); Donkey anti-Mouse IgG (H+L) Highly Cross-Adsorbed Secondary Antibody, Alexa Fluor 488 (Invitrogen, A21202); Donkey F(ab′)2 Anti-Rabbit IgG H&L, Alexa Fluor 647 (Abcam, ab181347); IRDye 800CW Secondary Antibody (LI-COR, 925-32211); and IRDye 680LT Secondary Antibody (LI-COR, 925-68020). The regents used in this study included Smoothened Agonist (Sigma-Aldrich, 566661); Digitonin (MCE, HY-N4000); FLAG Immunoprecipitation Kit (Millipore, FLAGIPT1); DAPI (Sigma-Aldrich, F6057); and isopropyl β-D-thiogalactoside (IPTG, Sigma-Aldrich, I6758).

### Animals.

*Osbpl2*^–/–^ mice were generated by Cyagen Biosciences. Briefly, 2 single-guide RNAs (sgRNA1: 5′-GGGACTCGGTGTAGCAGATA-3′, sgRNA2: 5′-TGAAATTGTGATAACAAGGC-3′) were designed to target the region flanking exons 3 and 4 in mouse *Osbpl2* gene. The mice carrying a heterozygous deletion of 3571 bp in the *Osbpl2* gene were identified and used for the subsequent work. Intercross heterozygous-targeted mice (*Osbpl2*^+/–^) were used to generate homozygous-targeted mice (*Osbpl2*^–/–^). Genotype analysis was performed using the primers showed in Supplemental Table 1. All mice were on the C57BL/6J background and were maintained under specific pathogen-free conditions at 22°C with a cycle of 12 hours of light/dark. Fresh water and rodent diet were available at all times.

### DPOAE and ABR measurements.

The DPOAE and ABR tests were performed in 1- to 6-month-old mice, as previously described (63). Briefly, mice (*n* = 6, 3 males and 3 females) were anesthetized with 4% chloral hydrate (0.1 mL/10 g) and then placed on a heating pad. The acoustic stimuli (5.6, 8, 11.3, 16, 22.6, and 32 kHz) were evoked using the Neuro-Audio (Neurosoft) system and TDT system (TDT). The ABR waveforms of evoked potentials were recorded from 90 to 0 dB with 5 dB sound pressure level (SPL) intervals, and the ABR peak V could be visualized at the same latency after an average of 1024 recordings. The hearing thresholds were determined by the lowest sound intensity that could induce a reproducible ABR waveform. The DPOAEs in response to 2 primary tones of frequencies f1 and f2 were recorded at (2 × f1) – f2, with f2/f1 = 1.2, and the f2 level was 10 dB lower than the f1 level. DPOAE thresholds were identified as the f1 level required to evoke a response at –10 dB SPL.

### Cell culture and treatment.

The HEI-OC1 auditory cells were derived from cochlear HCs of male mice, preserved by Federico Kalinec (The Regents of the University of California). *Osbpl2^–/–^* HEI-OC1 cells were generated by using CRISPR/Cas9–mediated gene editing. sgRNAs were designed using CHOPCHOP (http://chopchop.cbu.uib.no/) and were then recombined into pX330-U6-Chimeric_BB-CBh-hSpCas9 (AddGene). HEI-OC1 cells were transfected with CRISPR plasmid for 48 hours and treated with 2 μg/mL of G418 (Gibco) for 4–5 days to select positive clones. WT and *Osbpl2^–/–^* HEI-OC1 cells were cultured in Dulbecco’s modified Eagle’s medium (DMEM, Gibco) with 10% FBS (Gibco) at 33°C under 10% CO_2_. HEK293Ta cells were maintained in DMEM with 10% FBS at 37°C under 5% CO_2_. For gene overexpression, plasmids were transfected into cells using Lipofectamine 3000 (Thermo Fisher Scientific) according to the manufacturer’s instructions. Ciliogenesis was induced by serum starvation using the medium containing 0.2% FBS for 12–24 hours. SAG was added at 200 nM in starvation media for 12–24 hours to induce Shh pathway activation.

### Plasmids and DNA constructs.

pXJ40-HA, pXJ40-Flag, and pcDNA3.1-EGFP were purchased from the MiaoLing Plasmid Sharing Platform. Mouse *Osbpl2* and *Inpp5e* were amplified by PCR with Flag, HA, or EGFP tagged to the M-termini and cloned into plasmids. Flag-ΔFFAT and Flag-ΔORD constructs were made by deletion mutagenesis. 5HT_6_-HA-Inpp5e expression plasmid was constructed as previously described (39). The digested DNA encoding 5HT_6_ with 5′ and 3′ AgeI cleavage sites was subcloned into the HA-Inpp5e expression plasmid. All the constructs were verified by DNA sequencing. The primer sequences are listed in Supplemental Table 1.

### Immunofluorescence staining.

The cochleae were dissected from the temporal bone and fixed in 4% paraformaldehyde (PFA) in PBS pH 7.4 at 4°C overnight. The cochleae of P10 mice were placed in a 10% EDTA solution and decalcified for 3–4 days, which was not required for P1 or P3 mice. After removal of the covering bones, the basilar membrane was dissected from the cochlea as described (30), permeabilized, and blocked using 0.1% Triton X-100 with 10% normal goat serum. The basilar membrane was then incubated with the primary antibodies overnight at 4 °C, rinsed, and incubated with Alexa Fluor 488/555/647 conjugated secondary antibodies or phalloidin for 1 hour at room temperature. 

HEI-OC1 cells were grown on coverslips and fixed with 4% PFA for 15 minutes at room temperature followed by being permeabilized with 0.1% Triton X-100 and blocked in PBS containing 5% donkey serum. When performing immunofluorescence staining on PI4P and PI(4,5)P_2_, digitonin, instead of Triton X-100, was used for cell membrane penetration according to the manufacturer’s instructions. After incubation in blocks containing primary antibodies for 2 hours at room temperature or overnight at 4°C, the cells were incubated with appropriate secondary antibodies for 1 hour at room temperature. Subsequently, nuclei were counterstained with DAPI. The samples were visualized with a Zeiss LSM 700 confocal microscope. The acquired images were processed and analyzed by ZEN2 software (Carl Zeiss).

### Image processing and analysis.

Cilium position was determined by measuring the distance and angle of deviation from the cell center to the basal body and the ciliary length was measured using the line tool in ImageJ (NIH) (31). Cilia were outlined with the polygon tool, and the mean intensity of the desired channel inside the area was measured on an 8-bit scale. These values were then subtracted from the background and averaged for each field, which typically contained 30 or more cilia (51). The HC orientation was measured by the angle formed by the V-shaped stereociliary bundle and its deviation from the mediolateral axis of the epithelium, which were plotted in rose diagrams. The pooled data were obtained from 10–15 individual cells per row of HCs for 3 mice per genotype (or at least 50 cells from 2 independent cultures).

### RNA extraction and quantitative real-time PCR (qRT-PCR).

Mouse cochleae and HEI-OC1 cells were homogenized using the TRIzol Kit (Invitrogen) and total RNA was extracted according to the manufacturer’s instructions, of which 1 μg was used for cDNA synthesis via HiScript II One Step RT-PCR Kit (Vazyme). qRT-PCR was performed on a StepOne Plus system (Applied Biosystems) using ChamQ SYBR qPCR Master Mix. The relative expression levels of the selected genes were analyzed using the comparative CT method (2^−ΔΔCT^). Each qRT-PCR analysis was repeated at least 3 times and *Gapdh* was used as an internal control. Conventional qRT-PCR was carried out with the oligonucleotide primers shown in Supplemental Table 1.

### Immunoblotting.

Mouse cochleae and HEI-OC1 cells were lysed in cold RIPA buffer (Beyotime) with a protease and phosphatase inhibitor cocktail (MCE) on ice for 30 minutes. Samples were centrifuged at 12,000 *g* for 10 minutes, and the supernatants were stored at -80°C for later use. Protein concentrations were qualified by BCA protein quantification assay (Beyotime). A total of 50 micrograms of protein for each sample were loaded into 10% or 12% SDS–PAGE gels, which were prerun at 70 V and then at 100 V. The proteins were next transferred to PVDF membranes using the Trans-Blot Turbo transfer system (Bio-Rad). The membranes were blocked with 5% BSA or milk in tris-buffered saline containing 0.05% Tween-20 (TBST) (pH 7.4), incubated with the diluted primary antibody in 5% BSA at 4°C overnight, and then probed with the appropriate secondary antibody at room temperature for 1 hour. Detected proteins were visualized via the chemiluminescence method or performed with an Odyssey CLX Imaging System (LI-COR).

### Co-IP.

HEK293Ta cells expressing HA-Inpp5e and Flag-OSBPL2 were washed 3 times with cold PBS and then lysed with RIPA lysis buffer (Beyotime) containing complete protease inhibitors (MCE) for 30 minutes. The supernatant was collected and incubated with anti-FLAG M2 magnetic beads (Sigma-Aldrich) overnight at 4°C. The beads were washed with RIPA lysis buffer (Beyotime) at least 5 times. Immobilized protein complexes were eluted by denaturation in 5 × SDS loading buffer at 100°C for 10 minutes and then assayed by Western blot.

### GST pulldown assay.

pGEX-6p-1 plasmids, encoding GST, GST-OSBPL2, and pCzn1-His plasmid encoding His-INPP5E, were transformed in *E*. *coli* BL21 cultured with IPTG at 16°C for 18 hours. The cells were collected and lysed, and the total protein was purified with GST-tag Protein Purification Kit (Beyotime, P2262) and His-tag Protein Purification Kit (Beyotime, P2226) according to the manufacturer’s instruction. The proteins GST, GST-OSBPL2, and His-INPP5E were incubated with Glutathione Agarose (Thermo Fisher Scientific, G2879) overnight at 4°C and washed in IP Lysis Buffer (Beyotime, P0013) at least 3 times. Immobilized protein complexes were determined by Western blot.

### Statistics.

Statistical analysis was performed using an independent, 2-tailed Student’s *t* test in each case. GraphPad Software Prism 6 was used for plotting and all data were present as mean ± standard error (SEM). ANOVA was used to compare the means of multiple groups, and a *P* value less than 0.05 was considered significant.

### Study approval.

All animal experiments were approved by the Institutional Animal Care and Use Committee (IACUC) of Nanjing Medical University.

## Author contributions

XC, QW, and JY conceived the study and revised and approved the final paper. HS, HW, and CZ performed the experiments and analyzed the data. HS and HW wrote the first draft of the paper. YL, JY, ZC, and GX participated in data collection and interpretation.

## Supplementary Material

Supplemental data

## Version 1 01/18/2022

In-Press Preview

## Version 2 02/22/2022

Electronic publication
