# Supplementary material for: Mutations in OSBPL2 cause hearing loss associated with primary cilia defects via sonic hedgehog signaling
Source: JCI Insight. 2022 Feb 22;7(4):e149626. doi: 10.1172/jci.insight.149626 (PMC8876550; doi:10.1172/jci.insight.149626)
Supplement: Supplemental data [file jciinsight-7-149626-s196.pdf]

Supplemental Figures:

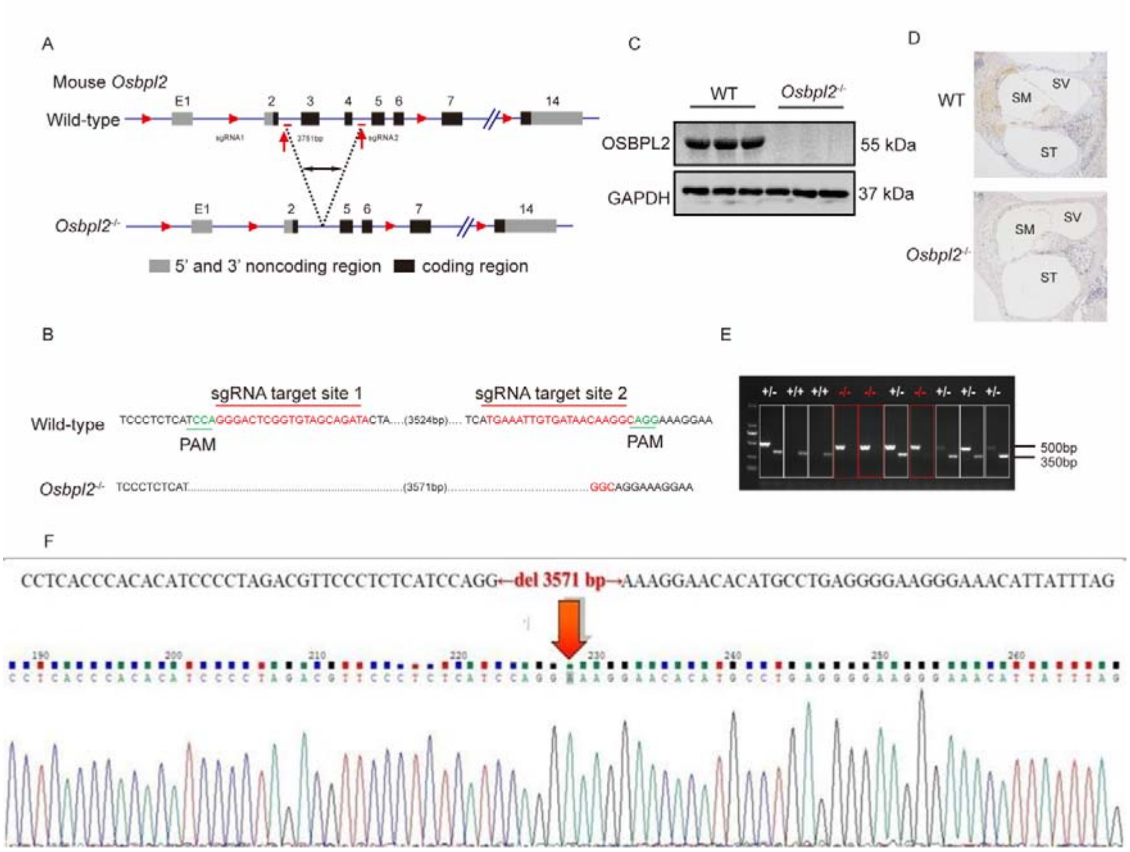

Supplemental Figure 1 Generation of *Osbpl2*$^{-/-}$ mice. (A) Schematic diagram of mouse *Osbpl2* gene. **(B)** SgRNAs were designed to target the region flanking exons 3 and exon 4 in mouse *Osbpl2* gene. SgRNA sequence (red), PAM domain (green). **(C)** Western blot analysis of OSBPL2 in cochleae of 1-month-old *Osbpl2*$^{-/-}$ mice (n=3). **(D)** Immunohistochemistry analysis of OSBPL2 in cochlea of 1-month-old *Osbpl2*$^{-/-}$ and WT mice. SV: scala vestibuli; SM: scala media; ST: scala tympani. **(E)** PCR analysis of genomic DNA from the tail biopsy of *Osbpl2*$^{-/-}$, *Osbpl2*$^{+/-}$ and WT mice. *Osbpl2*$^{-/-}$: 500 bp, *Osbpl2*$^{+/-}$: 500 bp /350 bp, WT: 350 bp. **(F)** The *Osbpl2*$^{-/-}$ mice carried a homozygous deletion of 3571 bp in mouse *Osbpl2* gene.

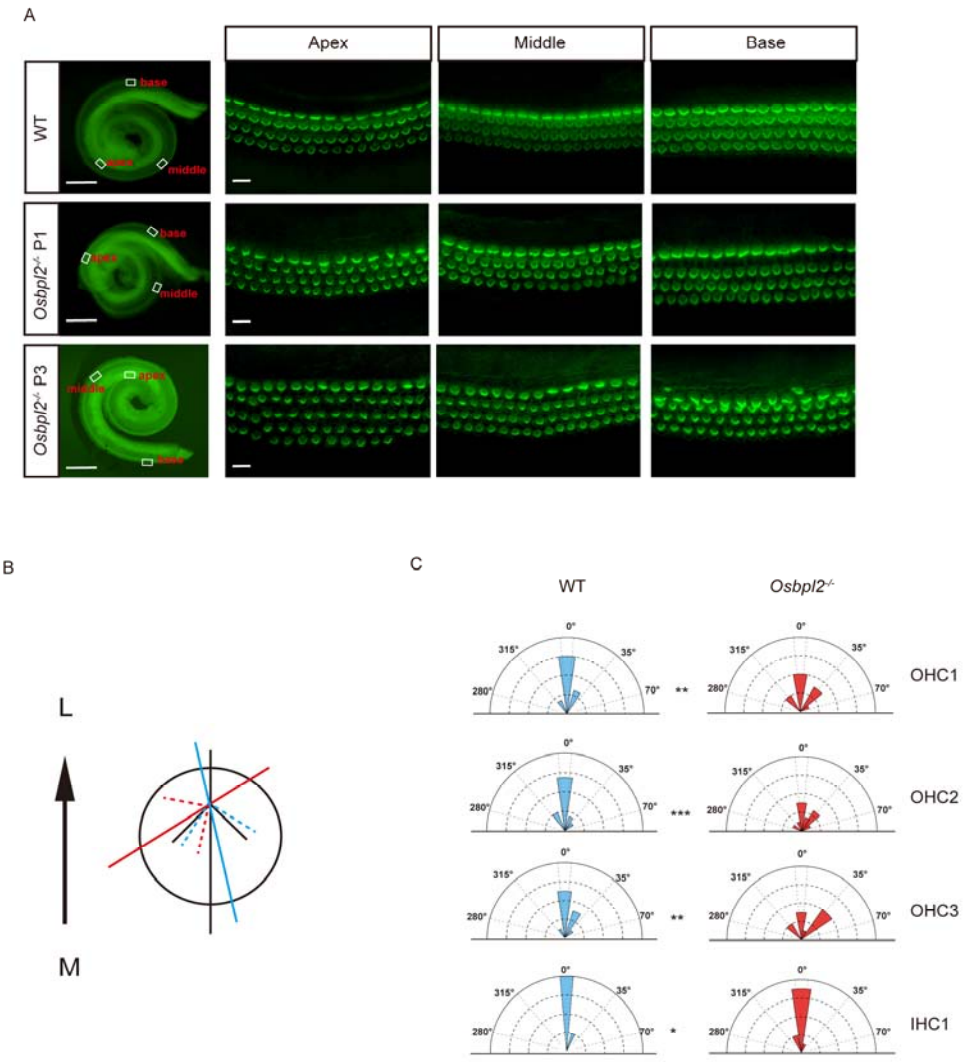

Supplemental Figure 2 Planar cell polarity (PCP) defects in *Osbpl2*$^{-/-}$ mice cochlea. (A) Immunofluorescence staining of sensory epithelium in *Osbpl2*$^{-/-}$ and WT mice (P1 and P3) with phalloidin (green). Scale bar: 5μm. **(B)** Schematic diagram of stereocilia bundles polarity. The HCs polarity was evaluated by measuring the angle between the stereociliary bundles and the medio-lateral axis of the epithelium. **(C)** Distribution of HCs orientation. The angle of deviation between the stereociliary bundle and the medio-lateral axis was plotted in rose diagrams (pooled data were obtained from 10-15 individual cells per row of HCs for 3 mice per genotype, **p*<0.05, ***p*<0.01 and ****p*<0.001 tested by Wilcoxon rank-sum test).

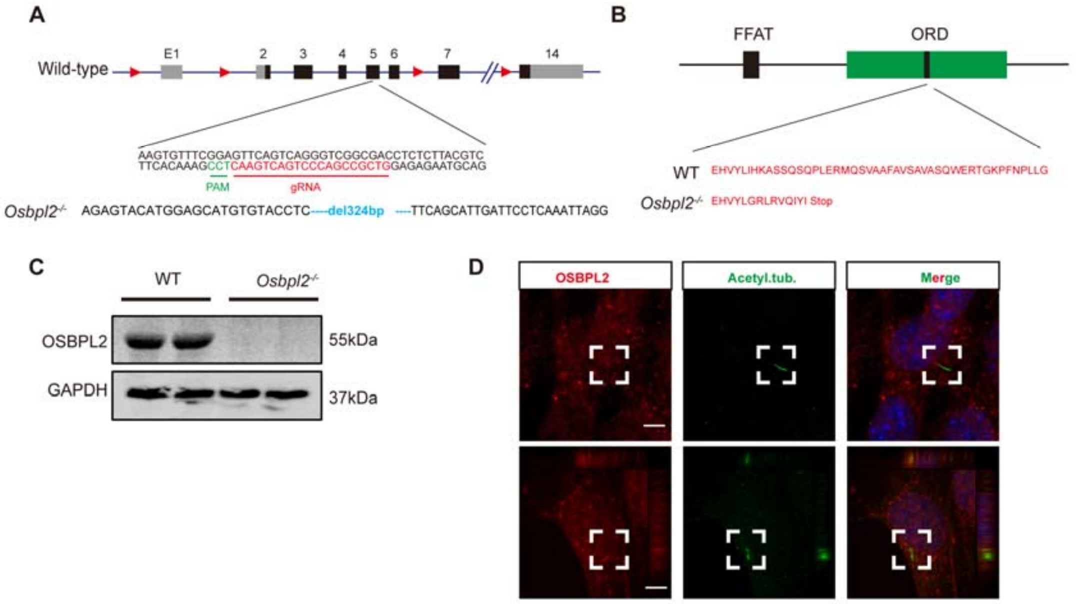

Supplemental Figure 3 Generation of *Osbpl2*$^{-/-}$ HEI-OC1 cells. (A) Schematic representation of the *Osbpl2*-knockout strategy. A biallelic mutation in *Osbpl2* gene (*Osbpl2*$^{-/-}$) was detected in a positive cell clone. **(B)** *Osbpl2*-knockout led to truncated protein. **(C)** Western blot analysis of OSBPL2 in *Osbpl2*$^{-/-}$ HEI-OC1 cells. **(D)** Immunofluorescence staining of the ciliary OSBPL2 in HEI-OC1 cells (serum-starved for 24 h) with anti-OSBPL2 (red), anti-acetylated tubulin (green) and DAPI (blue).

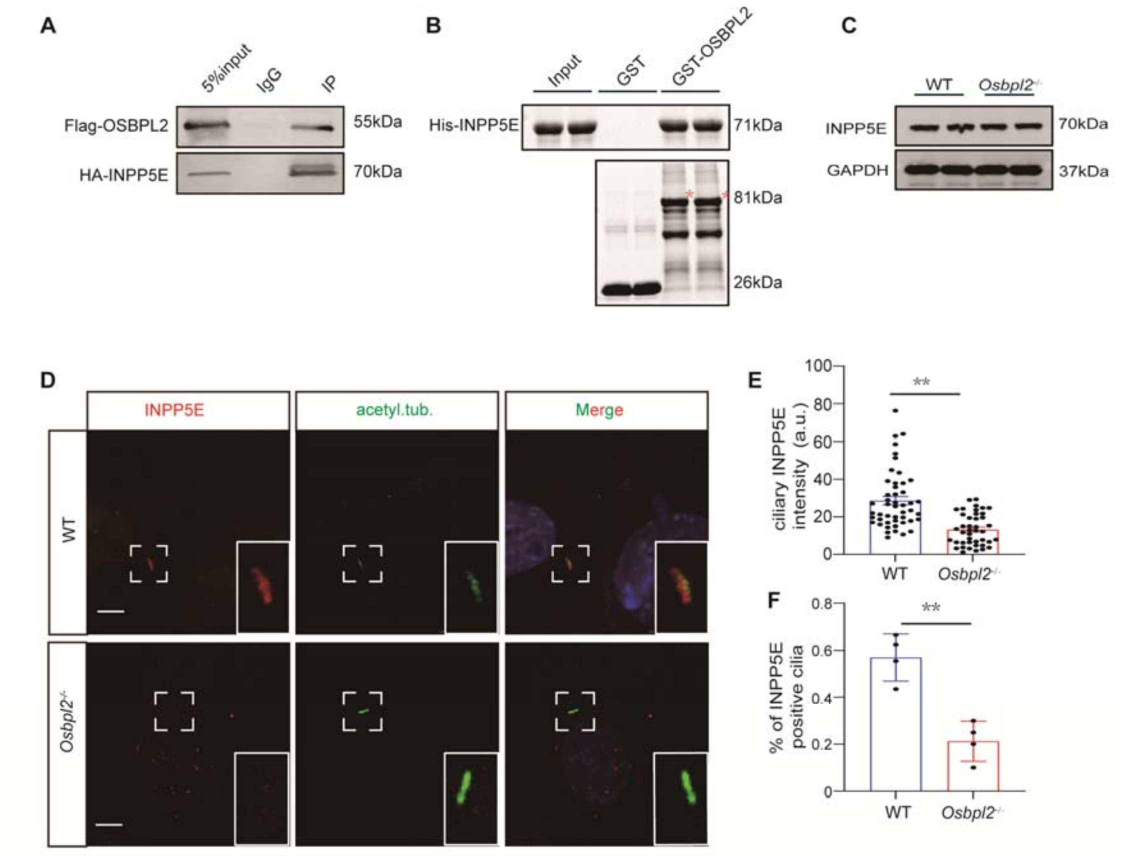

Supplemental Figure 4 OSBPL2 regulated the ciliary location of INPP5E. (A) Co-IP assay verified the interaction of OSBPL2 with INPP5E. HEK293Ta cells transfected with Flag-OSBPL2 was used as negative control. **(B)** *E. coli* expressing His-INPP5E was used in GST pulldown assays, which verified the interaction of OSBPL2 with INPP5E. **(C)** Immunoblot analysis of INPP5E in whole cell lysate of *Osbpl2*$^{-/-}$ and WT HEI-OC1 cells. GAPDH served as a loading control. **(D)** Immunofluorescence staining of the ciliary INPP5E in *Osbpl2*$^{-/-}$ and WT HEI-OC1 cells (serum-starved for 24 h) with anti-INPP5E (red), anti-acetylated tubulin (green) and DAPI (blue). Dashed frames denote the locally zoomed regions in solid frames (bottom right). Scale bar: 5μm. **(E)** Quantification of ciliary INPP5E intensity in *Osbpl2*$^{-/-}$ and WT HEI-OC1 cells (at least 30 cells from a microscope field, each dot represents a cell. ***p*<0.01 tested by Student's *t* test). **(F)** The proportion of INPP5E positive cilia in *Osbpl2*$^{-/-}$ and WT HEI-OC1 cells (at least 30 cells from a microscope field, each dot represents the proportion of INPP5E positive cilia in a microscope field. ***p*<0.01 tested by Student's *t* test).

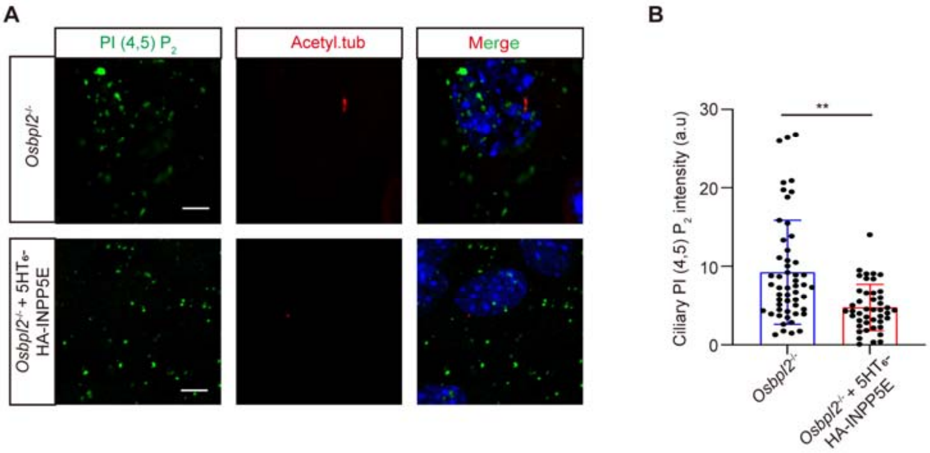

Supplemental Figure 5 The accumulation of PI(4,5)P2 at the base of the cilia was alleviated by INPP5E expression. (A) Immunofluorescence staining of the ciliary PI(4,5)P2 in *Osbpl2*$^{-/-}$ HEI-OC1 cells expressing 5HT6-HA-INPP5E. Cells was stained with anti-acetylated tubulin (red), anti-PI(4,5)P2 (green) and DAPI (blue). Scale bar: 5μm. **(B)** Quantification of ciliary PI(4,5)P2 intensity in *Osbpl2*$^{-/-}$ HEI-OC1 cells expressing 5HT6-HA-INPP5E (at least 30 cells from a microscope field, each dot represents a cell. **p<0.01 tested by Student's t test).

Supplemental Table Captions

Supplemental Table 1 Primers for PCR-based sequencing and qRT-PCR

Table 1 Primers for PCR-based sequencing and qRT-PCR

**Sequence Name**	**Sequence (5' to 3')**
HA-*Inpp5e*-F	GTGCCAGACTACGCAGGATCCATGCCATCCAAGTCAGCT
HA-*Inpp5e*-F	TTTAATAAGATCTGGTACCTCAGGACACGGTGCAAACT
Flag-*ORP2*-F	CGACGATGATAAGTCCGGATCCATGAACGGGGAGGAAGA
Flag-*ORP2*-R	TTTAATAAGATCTGGTACC GTATATGTCTGGGCAGT
$5HT_6$-HA-F	ATGGTTCCAGAGCCCGGC
$5HT_6$-HA-R	TCAGTTCATGGGGGAACC
Flag-△ORD-F	CGACGATGATAAGTCCGGATCCATGAACGGGGAGGAAGAAT
Flag-△ORD-F	TTTAATAAGATCTGGTACCTTAACTTTTGCTGGTATCCAAGT
Flag-△FFAT-F	CGACGATGATAAGTCCGGATCC ATGACTAGGA
Flag-△FFAT-F	TTTAATAAGATCTGGTACC GTATATGTCTGGGCAGT
q-M-*Gli1*-F	CCAAGCCAACTTTATGTCAGGG
q-M-*Gli1*-R	AGCCCGCTTCTTTGTTAATTTGA
q-M-*Ptch1*-F	AAAGAACTGCGGCAAGTTTTTG
q-M-*Ptch1*-R	CTTCTCCTATCTTCTGACGGGT
q-M-*Gapdh*-F	AGGTCGGTGTGAACGGATTTG
q-M-*Gapdh*-R	TGTAGACCATGTAGTTGAGGTCA
Osbpl2-OC1-F	CAAATCGGAACTGGAGGAAC
Osbpl2-OC1-R	CCCCGGCCTGGTACACTG
Osbpl2-mice-F1/F2	TATAGTGGACTTACCGAAATCTCAG
Osbpl2-mice-R1-KO	TGGCTACTAGACACTCCATTTC
Osbpl2-mice-R2-WT	CATCAATAAGGCTGAAGCCATC
